# Visualization and 3D Reconstruction of Flame Cells of *Taenia solium* (Cestoda)

**DOI:** 10.1371/journal.pone.0014754

**Published:** 2011-03-11

**Authors:** Laura E. Valverde-Islas, Esteban Arrangoiz, Elio Vega, Lilia Robert, Rafael Villanueva, Olivia Reynoso-Ducoing, Kaethe Willms, Armando Zepeda-Rodríguez, Teresa I. Fortoul, Javier R. Ambrosio

**Affiliations:** 1 Departamento de Microbiología y Parasitología, Facultad de Medicina, Universidad Nacional Autónoma de México (UNAM), México City, Distrito Federal, México; 2 Departamento de Biología Celular y Tisular, Facultad de Medicina, Universidad Nacional Autónoma de México (UNAM), México City, Distrito Federal, México; 3 Departamento de Visualización, Dirección General de Cómputo y de Tecnologías de Información y Comunicación (DGTIC), Universidad Nacional Autónoma de México (UNAM), México City, Distrito Federal, México; 4 Laboratorio de 3D, Facultad de Medicina, Universidad Nacional Autónoma de México (UNAM), México City, Distrito Federal, México; The George Washington University Medical Center, United States of America

## Abstract

**Background:**

Flame cells are the terminal cells of protonephridial systems, which are part of the excretory systems of invertebrates. Although the knowledge of their biological role is incomplete, there is a consensus that these cells perform excretion/secretion activities. It has been suggested that the flame cells participate in the maintenance of the osmotic environment that the cestodes require to live inside their hosts. In live Platyhelminthes, by light microscopy, the cells appear beating their flames rapidly and, at the ultrastructural, the cells have a large body enclosing a tuft of cilia. Few studies have been performed to define the localization of the cytoskeletal proteins of these cells, and it is unclear how these proteins are involved in cell function.

**Methodology/Principal Findings:**

Parasites of two different developmental stages of *T. solium* were used: cysticerci recovered from naturally infected pigs and intestinal adults obtained from immunosuppressed and experimentally infected golden hamsters. Hamsters were fed viable cysticerci to recover adult parasites after one month of infection. In the present studies focusing on flame cells of cysticerci tissues was performed. Using several methods such as video, confocal and electron microscopy, in addition to computational analysis for reconstruction and modeling, we have provided a 3D visual rendition of the cytoskeletal architecture of *Taenia solium* flame cells.

**Conclusions/Significance:**

We consider that visual representations of cells open a new way for understanding the role of these cells in the excretory systems of Platyhelminths. After reconstruction, the observation of high resolution 3D images allowed for virtual observation of the interior composition of cells. A combination of microscopic images, computational reconstructions and 3D modeling of cells appears to be useful for inferring the cellular dynamics of the flame cell cytoskeleton.

## Introduction

Flame cells (FC) are ciliated cells located within the basal matrix, at the neodermal tissue of cestoda. They are considered as terminal cells in flame or bulbs [1] and as the basic units of the protonephridial system (PS) of invertebrates. In cestodes, like in all parasitic platyhelminthes, the PS are the excretory systems with an important role that allow the parasites to conserve water and eliminate salts and survive in the intestine or body cavities of their hosts and because of that, they act like osmoconformers [2], [3]. Parasites need to maintain, within physiological limits, the osmotic pressure of their tissues against that of the host environment [2].

FC have a typical morphology: by light microscopy they look like comets in which the anterior end of the cell corresponds to the cell body while the distal end contains the tuft of cilia known also as the flames [1]. By transmission electron microscopy (TEM), FC of several helminthes exhibits a similar morphology [1], [3]: a single nucleus and classical cilia with 9+2 axonemes. The plasma membrane surrounds the cell body that extends through the tip of the tuft of cilia and is apparently in close interdigitation with the membrane of adjoining cells [4]. While the bodies of FC are immersed in the parenchymal tissue, the cilia tufts are extended inside the PS tubules [3], [5]. In tapeworms, FC appear to be beating continuously, their cilia producing the impression of flickering flames inside the cell [4], and due to this behavior, FC have also received names like cap cells and bulb cells [5]. Knowledge of cytoskeletal proteins of FC have shown that polymerized actin was found by fluorescent phalloidin in FC of the cestode *Diphyllobothrium dendriticum* [6] and in the monogenean *Gyrodactylus rysavyi* [7] where it was associated to longitudinal fibers that are in close contact with the PS excretory system. Tubulin was demonstrated in FC cilia of the cestode *Gymnorinchus* [8] and in the trematode *Schistosoma mansoni* [9]. However, the knowledge of the FC morphology, their role in the parasite physiology is scarce and there is no clear evidence of their functional activities [1]. It is mainly assumed to be related to excretory activities trough the PS.

In tapeworm infections, as those produced by the metazoan *T. solium*, the FC of the parasites appear to be important for the survival within hosts because they can perform specific functions such as detoxification or transformation of substances harmful to the parasites [2], [3]. It is well known that parasitic stages of the medically important of *T. solium* (the larval stage or cysticercus and the adult stage or taenia) have many FC in their tissues and the morphology of these cells has only been demonstrated in invaginated cysticerci of *T. solium* stained by Bodiańs protargol method [10], [11]. The importance of study of these cells is that they could be excellent targets of the antihelminthic treatments based on their capacity to inhibit the polymerization of microtubules using benzimidazole drugs such as albendazole, as shown after the *in vitro* treatment of cysticerci of the murine model of *T. crassiceps* [12].

The aim of the present work is to define the cytoarchitecture of *T. solium* FC with the help of microscopic observations, digital reconstructions and 3D visualization of the cytoskeleton of these cells that could be useful as an alternative way of deducing the functions of microscopic structures of ciliated cells. In addition, we consider that increasing knowledge of the function of the *T. solium* FC could extend our understanding of the cellular biology and physiology of these cestodes, which are still endemic in many countries [13].

## Materials and Methods

### Parasites

Larvae were dissected from muscles of naturally infected pig meat, extensively washed with cold phosphate buffered saline (PBS) adjusted to pH 7.2 and divided in groups either for microscopic observation or for inducing experimental infections in golden hamsters. Intestinal adult parasites were obtained from immunosuppressed hamsters 30 days after oral administration of cysticerci as reported previously [14].

This study was approved by the local ethics and research committees of the Facultad de Medicina, UNAM (FMED/CI/RGG/022/08/2006, FMED/CI/RGG/055/08/2007 and 144-2009.)

### Microscopy

Live-Cell Microscopy. Live cysticerci were maintained for one or two days *in vitro* in RPMI 1640 medium supplemented with 25 mM HEPES buffer adjusted to pH 7.2 and 30 mM carbonate salts. Parasites were maintained in a humidified incubator at 37 °C in a 5% CO_2_ environment.

For filming motion of FC in live parasites, cysticerci were punctured with a needle in order to eliminate the vesicular fluid and observations were performed on the internal side of the bladder walls, by spreading the tissue on a microscope slide and observing it directly using Nomarsky differential-interference-contrast microscopy (DIC). Time-lapse recordings of FC dynamics, at room temperature (RT), were carried out as follows: Each frame was captured at the rate of 3.2 s/frame producing a total of 34 frames using a LSCM Leica.

### Processing of parasites for fluorescent assays

Parasites were processed by standard procedures for immunofluorescence assays. Briefly, parasites were washed in phosphate-buffered-saline (PBS), embedded in Tissue-Tek (Sakura, USA), sectioned (10 µm) in a cryostat (CM Leica 1100) and sections adhered on poly-L-Lys treated glass slides. Tissues were fixed with cold acetone and washed with PBS and PBS/0.3% Tween-20. Blocking of sections was done with PBS/0.3% Tween-20/2% BSA and finally incubated for 1 h in the presence of anti-α-tubulin DM1-A (Sigma, Missouri, USA) (1∶100), as a primary antibody and with anti-mouse IgG conjugated to FITC (ZYMED, California, USA) as secondary antibody (1∶30). F-actin was located using Alexa fluor 594 Phalloidin (Invitrogen, Molecular Probes, Oregon, USA) (1∶40) diluted in the solution that contained the secondary antibody. Myosin II was revealed by an indirect immunoassay; as a primary antibody, a polyclonal anti-*T. solium* myosin II antibody was prepared as previously published [14], [15]. As secondary antibody, an anti-rabbit antibody coupled to biotin and finally, for developing of the fluorescent red color, a rhodamine coupled to avidine was used (Vector Laboratories (Burlingame, CA, USA). Nuclear DNA staining was performed by adding Propidium Iodide (Sigma, Toluca, México) (1∶1000) or DAPI (Sigma, Toluca, México) (1 mg/ml) 5 min before the slides were examined. For observation, slides were washed with PBS and mounted in a commercial mounting solution for preserving fluorescence (Dako, CA, USA). Control observations were done on parasite cryosections only incubated with secondary antibodies. Unless described, all reagents were from Sigma.

### Microscopic fluorescence observations

In order to obtain a photographic composition of sections from a whole cysticercus, immunofluorescent images were taken and processed as indicated by us during the evaluation of the tisular distribution of actin in *T. solium* [16].

### Confocal microscopy

Time-lapse recordings were performed using a LSCM Leica (TCS-SP5) equipped with PlanFluor DIC objective lens (40× 0.75 NA). The hardware was driven by Leica Microsystems 1.8.0. Hardware and compilation of image sequences are described below in conjunction with confocal microscopy observations.

Co-localization of fluorescence was performed using three different LSCM equipments:

1. LSM 5 PASCAL (Zeiss) equipped with Argon-Krypton and Helium-Neon lasers using filters BP 450–490 and BP 546/12 at magnifications of 20× (0.5 NA), 40× (1.3 NA oil-D16) and 100× (1.3 NA oil pol) using PLAN NEO FLUOR objective lenses.
2. FV1000 (Olympus) at magnifications of 20× (0.75 NA), 40× (1.25–0.75NA), 60× (1.35 NA) and 100× (1.40 NA) using UPLSAPO objective lenses.
3. Fluorescence microscope AXIOVERT (Axioplan 2 Imaging Zeiss) adapted with an Apotome system. For recording, analyzing and converting images to TIFF formats, images were processed using specific computational packages of each equipment: LSM 5 PASCAL Version 2.8, FV10-ASW 1.4 and Axiovision V4.1. In LSCM equipments, Z sections were obtained after adjusting the beams for recovering 15–20 sections of ∼0.5 µm thickness.

For LSCM equipments, laser beams were adjusted to wavelengths for emission and excitation of fluorescein and rhodamine fluorophores at 488, 633 and 520, 590 nm, respectively.

### Electron Microscopy

Scanning electron microscopy (SEM) of adult parasites was performed as described for *Fasciola hepatica* [17] using a DSM-950 Zeiss equipment adjusted to 25 kV.

Transmission Electron Microscopy (TEM) of cysticerci and adult parasites was performed using samples embedded in Lowicryl [18] and in Spurr resins [19], where thin sections (40–80 nm) were obtained in a microtome (Leica), mounted on 300 mesh formvar covered nickel grids and examined in a JEOL (JEM-1200 EXII) at 60–70 kv.

Images from SEM and TEM were recorded on Kodabrome II RC films (Kodak F2 and F5, Rochester, New York) and were scanned and processed as indicated below.

Immuno-electron TEM microscopy was followed as described in [18]; α-tubulin was detected using DM1-A antibody as a primary antibody (1∶100) and commercial anti-mouse IgG coupled to 20 nm colloidal gold (BBInternational, UK) as a secondary antibody (1∶100).

### Processing of images

For TEM and SEM microscopical observations, micrographs were scanned in a HP LaserJet 3050 Scanner 3060 and contrast adjustment was conducted using Adobe Photoshop V.7.0. Compilation of images from LSCM was processed as described below.

### Computational processing of fluorescent images

Tridimensional reconstruction, 3D visualization and virtual imaging. After recovering stack Z plain sections, the images were stored in different formats depending on the microscope used: TIFF for Axiovert, Leica and Olympus and MLS for Zeiss. Images were then processed in a SGI Onyx 350 computer localized at the IXTLI Visualization Observatory (DGTIC, UNAM). Reconstruction of stack images was performed using AMIRA V3.1 software where 3D images were produced after creating polygonal surface models with different colors. 3D images were stored using TIFF and MPEG formats.

3D dynamic animations. After FC reconstructing by AMIRA software, the interactive images of virtual morphology, structure and functionality of FC were produced at the 3D Laboratory (Facultad de Medicina, UNAM) using a 3DS MAX software V8 (AUTOCAD).

## Results

### Live-Cell Microscopy

As seen in the supplemental movie S1, FC exhibit continuous movements, they are embedded in the interstitial matrix (IM). At 40× magnification with Nomarsky optics, in one single selected cell, the continuous ciliary tuft flickering was observed inside of an excretory duct.

### Fluorescence Microscopy

**Figure 1:**
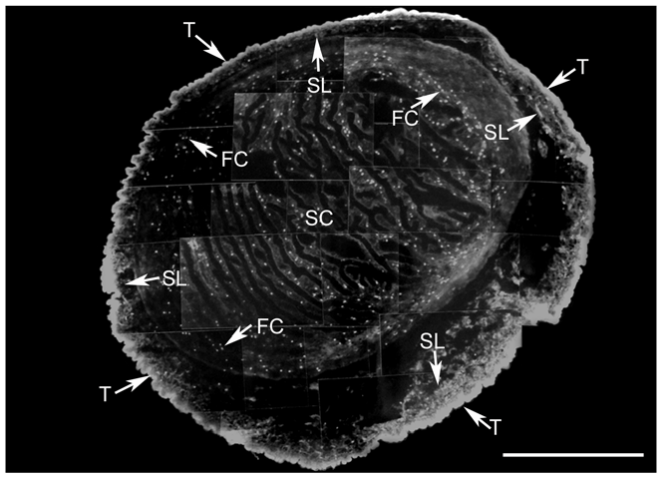
*T. solium* larval stage α-tubulin distribution. A photographic composite of images from cysticercus cryosections reveals the localization of α-tubulin using DMI-A mAb as a primary antibody. The fluorescein coupled to the secondary antibody was distributed along the tegumentary wall (T), the subtegumentary layer (SL) and in dots corresponding to flame cells (FC) that were mainly scattered in the folds of the invaginated scolex (SC). Scale bar = 1 mm.

**Figure 2:**
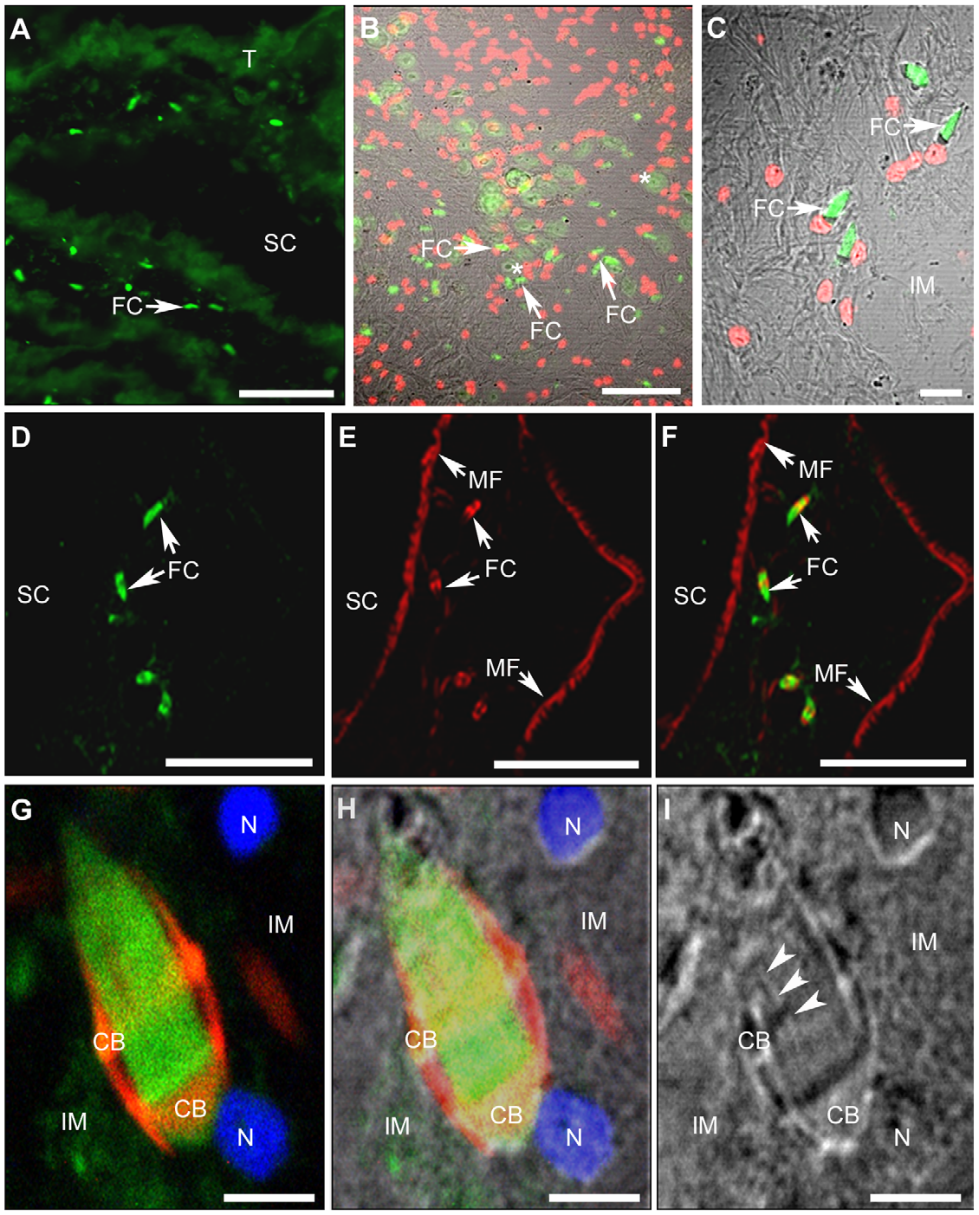
α-tubulin and F-actin in flame cells. α-tubulin (green) and F-actin (red) were revealed in flame cells (FC) of the invaginated scolex (SC). Upper panel: α-tubulin in flames (A–C) and nuclei of the cells stained red with propidium iodide (B and C). In B, under Nomarsky illumination, several FC are seen surrounding protonephridial ducts (*). Middle panel: α-tubulin in flames (D, F) and F-actin in FC and myofibers (MF) (E, F). Button panel: A higher magnification (60×) of a cell (G, H) shows the different distribution of α-tubulin and F-actin: the former is in the flame and the later is surrounding an anterior portion of the flame like a belt clasp (CB). Nuclei were stained with DAPI (blue). As seen under Nomarsky illumination (H, I), the cell is embedded in the interstitial matrix (IM) and several cilia can be distinguished (arrowhead) in its ciliary tuft. Scale Bars A and B = 50 µm, C = 10 µm, D–F = 40 µm and G–I = 2.5 µm.

By epifluorescence, recognition of DM1-A antibody was found to have a wide distribution in *T. solium* cysticerci tissues, where it was located in the tegument and less in the subtegumental layer. At the invaginated scolex, with low magnification (20×), the DM1-A antibody was found to react with many fluorescent dots scattered mainly in the tissues surrounding the spiral canal (Figure 1). With increased magnification, (Figure 2) several fluorescent dots, surrounding muscular fibers or interstitial matrix were revealed (Figure 2, top and middle panels). The dots were seen to be elongated (Figure 2A) and in order to see if they corresponded to FC it was decided to stain all nuclei with PI and to co-localize them with fluorescent secondary antibodies against DM1-A as shown in the spiral canal (Figures 2B and 2C, top panel). In the middle panel of the figure, DM1-A and phalloidin were found (Figures 2D–2F in green and red, respectively) each with a distinct distribution inside of FC. F-actin and α-tubulin, the recognized proteins, were mainly expressed in specific cellular structures of the cells as presented in the bottom panel of the Figure 2: F-actin was found with a distribution that resembled a belt clasp in the FC, in addition to parenchymal muscular fibers situated in close vicinity to these cells (Figure 2E and 2F, middle panel), while α-tubulin was found in the tufts of cilia (indicated by arrowheads in the corresponding images). Apparently, belt clasps of FC are surrounding the α-tubulin of the ciliary tufts (Figure 2G and 2H, bottom panel).

**Figure 3 pone-0014754-g003:**
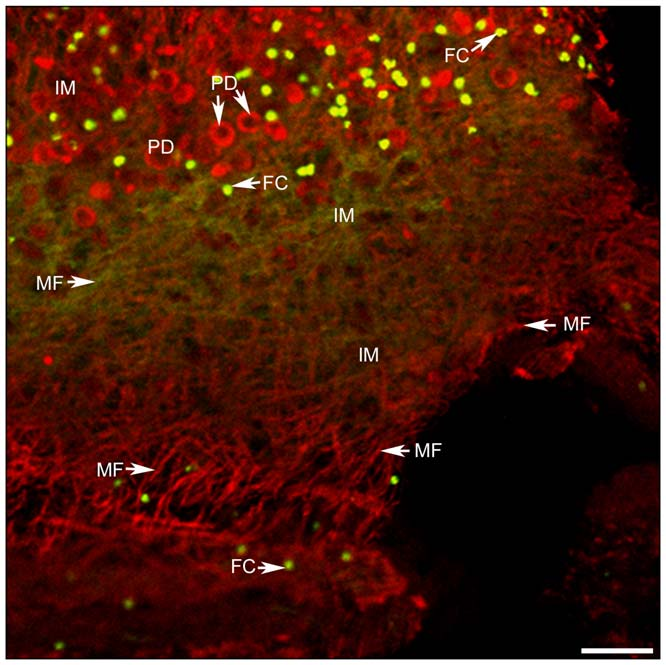
F-actin and myosin II in the invaginated scolex. F-actin (green) is shown in green as done in figure 2. Myosin II was revealed in red. A selected field of the invaginated scolex there is a network of myofibers (MF) that contain F-actin or myosin II. Myosin was also found surrounding several cross-sectioned protonephridial ducts (PD) that, in turn, are surrounded by clusters of bright fluorescent green dots, that correspond to the co-localization of F-actin and myosin II on the belt clasps of flame cells (FC). Some FC appear encrusted in myofiber bundles and in the interstitial matrix (IM). Scale bar = 24 µm.

Fluorescent staining for F-actin and myosin II (Figure 3 where F-actin is in green due to green phalloidin and myosin II in red due to rhodamine-avidine) revealed that both cytoskeletal proteins were co-localized at many high fluorescent yellow dots that are near to circular structures and embedded in the interstitial matrix. Localization of fluorescent markers was different in myofibers and rhodamine-avidine was found to be concentrated at circular structures of similar size as those described as unstained circular holes at Figure 2B.

### 3D reconstruction and visualization of flame cells

**Figure 4 pone-0014754-g004:**
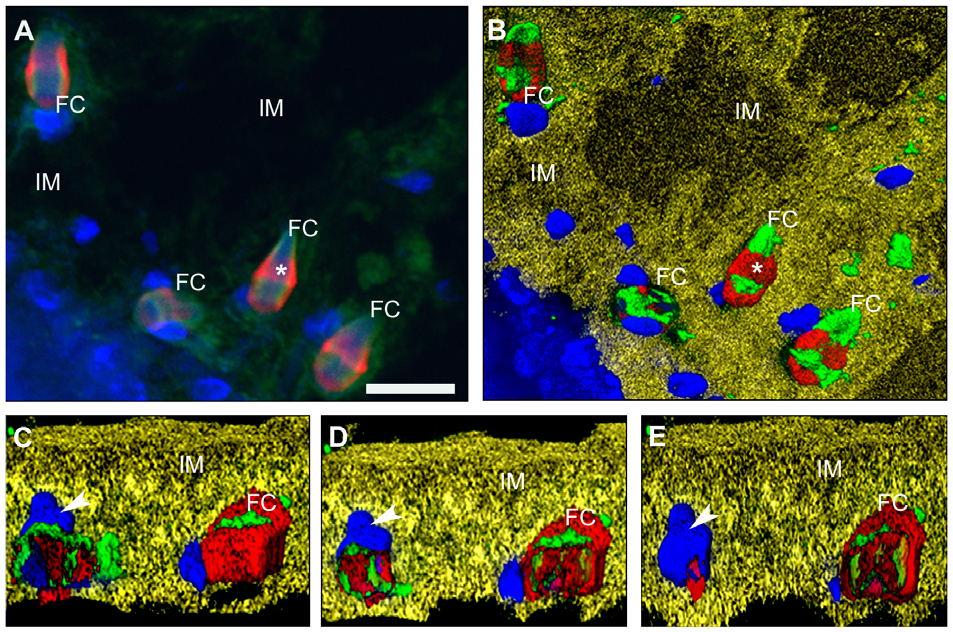
Reconstruction and 3D visualization of flame cells. A) Fluorescent staining of F-actin, α-tubulin and nuclei as indicated in Figure 2F. B) A reconstruction and visualization of the image in A after rendering and reconstructing by AMIRA software a stack of ∼20 images. False colors, similar to those seen by fluorescence, were associated to cytoskeletal proteins and nuclei of flame cells (FC) and, in order to reconstruct the interstitial matrix (IM), a yellow color was selected. Rotation (90°) of the focal plane of FC (C–E), after digital cross sectioning of the image in B, is showing that inside of the clasp the F-actin is forming a cage. 3D visualization of the button panel shows that FC are embedded in tunnels formed in the matrix (see Movie S5). Scale bar = 10 µm.

Having established that fluorescent probes were found in specific structures of the cytoskeleton of FC, we processed the Z image stacks in order to obtain a 3D reconstruction as shown in movies S3 and S4. In these movies, we have centered our attention in the reconstruction of the actin clasp and according with the observations, the structure could be hollow inside (Movie S3) and its reconstruction presents the complete spatial volume of this intracellular structure (Movie S4) as seen at the DIC Figure 2I of the button panel (indicated as CB). In Figure 4 there is a comparison between a selected image acquired trough LSCM (Figure 4A) and how it appears after a 3D reconstruction (Figure 4B) and visualization (Figure 4C–E); In the 3D image, the original fluorescent colors (Figure 4A) were preserved: nuclei in blue, α-tubulin in green and F-actin in red while in the 3D visualization it was possible to include the reconstructed IM in yellow color (Figure 4B). Four FC were observed, one of them is displayed in a different orientation; the flame is projected to the observer. Other FC are in a longitudinal plane and exhibit all the cellular structures described in Figures 2G and 2H. In the Figure 4C–E, two FC are seen, however one of them is apparently in a different plane, projecting the ciliary tuft toward the observer (Indicated by an arrowhead in the Figure). Movie S5 corresponds to the reconstruction of FC seen in Figure 4B where the ciliary tufts are apparently oriented to the lumen of an unstained PD. As the reconstruction of the FC is shown in the movie S5; the actin clasp is clearly surrounding the flame of the cell in a transverse section as also seen in Figures 4C–4E.

In order to obtain digital cross sections of the cells focusing from the nuclei to the tip of the cilia, a rotation of the 3D visualization image was performed as shown in the sequential images of Figure 4 (4C–E). At the level of the actin clasp, digital sequences indicate that this structure is embracing the FC as previously described and was shown in the movie S4. Inside the clasp was found a hollow place which presumably corresponds to the space shared by the cytoplasm and the beginning of the ciliary tuft (in the Figure 4C, in the FC on the right, the green color inside and behind the clasp is associated to the tip of the tuft) as seen in the movie S3. In digital sections of images 4C to 4E, the nucleus of one FC is diminishing in size until the remaining blue color appears as a small blue dot. IM in yellow and the FC on the left, loses the body to leave only the blue nucleus.

### Ultrastructural Observations

**Figure 5 pone-0014754-g005:**
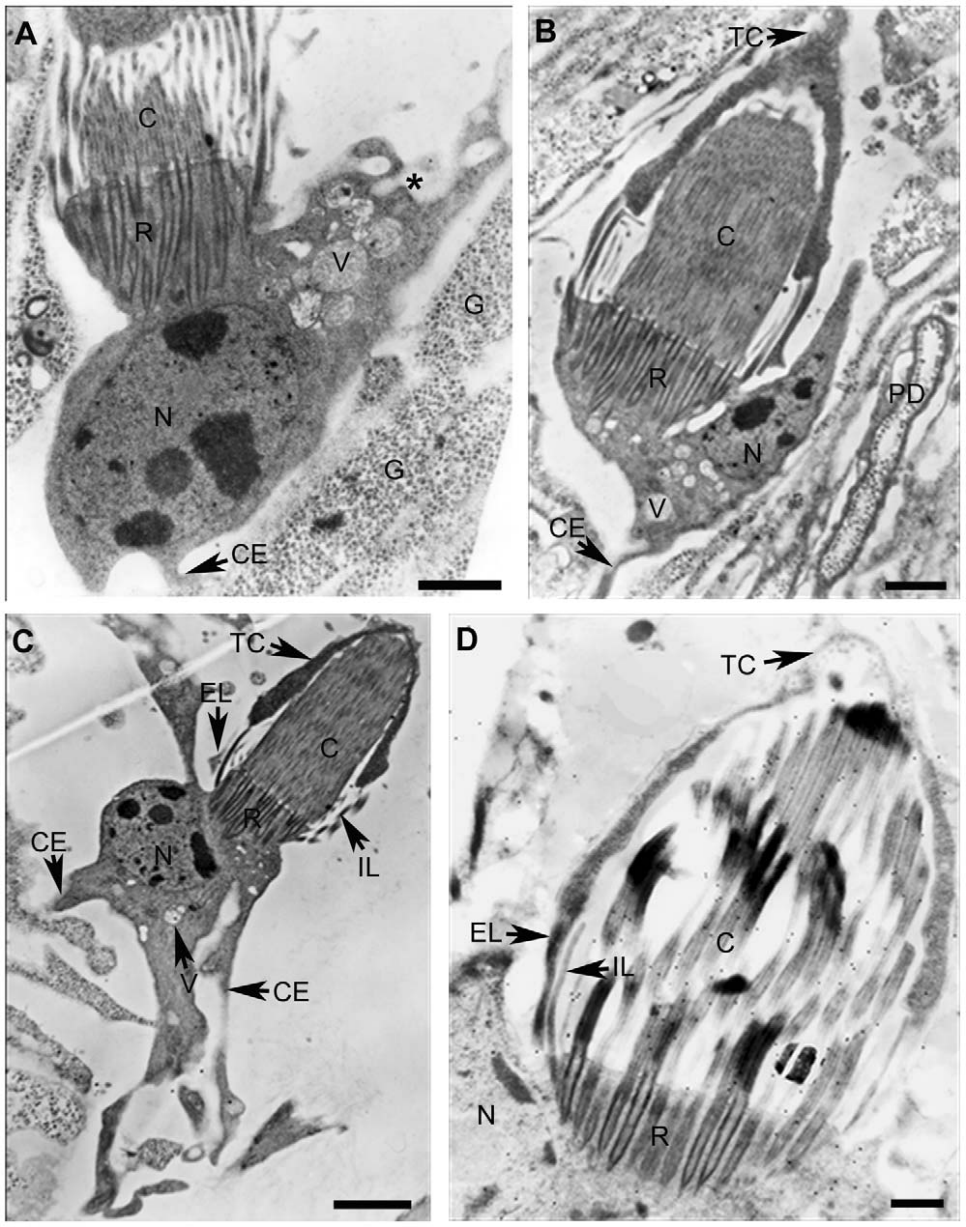
Transmission electron microscopy of flame cells. The complete morphology of three ciliated cells (A, B and C) shows their cell bodies attached to the flames trough a structure of cross-striated rootlets (R). In cell bodies, nuclei (N) are large and have several heterochromatic regions. Cytoplasmic regions have microvesicles (V) empty, filled or discharging through membrane invaginations (*) and some cytoplasmic extensions (CE) appear connected with a glycogen storage cell (G). Interactions between FC and TC (Figures B–D) are due to two external (EL) and internal leptotriches (IL). α-tubulin in cilia of the flame is shown because of the distribution of 20 nm gold conjugated secondary antibody after its interaction with DM1-A mAb (D). Observations were performed using a JEOL 1200EX TEM at 60.0 KeV. Scale bars A and B = 1 µm, C = 2 µm and D = 500 nm.

**Figure 6 pone-0014754-g006:**
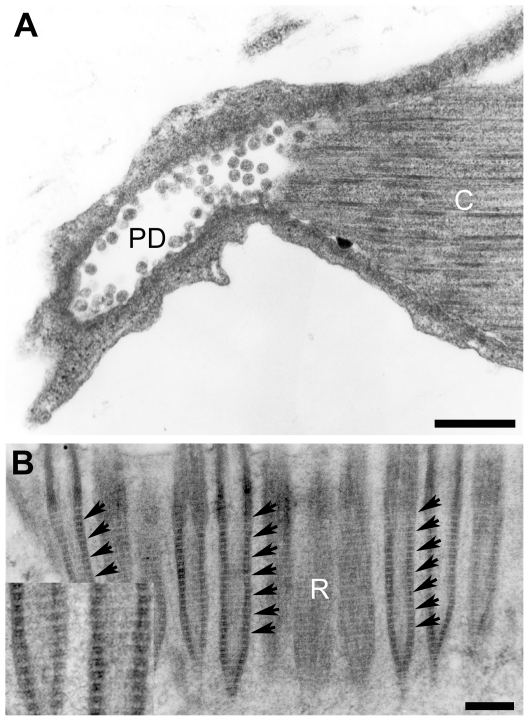
Ultrastructure of the protonephridial duct and a cross-striated rootlet of a flame cell. In A, the lumen of a protonephridial duct (PD) contains small rounded electrodense vesicles presumably coming from cilia (C). In B, at the cross-striated rootlet (R), cilia are bound by a fine regular network of microfibrils (small arrows) that cross the region from one side of the FC to the other. The insert is showing a digital magnification of a selected adjacent region of the correspondent basal bodies. Scale bars A = 500 nm and B = 200 nm.

Several FC were observed by TEM as shown in Figures 5 and 6. In longitudinal sections, cells exhibit the ciliary tuft and a cell body containing the nucleus and microvesicles; Cytoplasmic extensions or pseudopods were observed (Figure 5A–C); At the level of the ciliary tuft, extensions of the cytoplasm, coming from the cross-striated rootlets, were extended along the cilia until they interact with the plasma membrane of the protonephridial duct cells and some internal leptotrichs were seen. Inside of the ciliary tuft a bunch of long cilia (Figure 5C) were found to be connected to collecting ducts of cells of the protonephridial system as seen in the magnification showed in Figure 6A. α-tubulin was detected by immunogold decoration in ciliary microtubules (MT) (Figure 5D). The region of the FC that it is not associated with the PD could correspond to the actin clasp structure seen by fluorescence microscopy (Figure 2).

Closer observation of intracellular structures shows nuclei with euchromatin and several heterochromatin regions (Figure 5A–C). In the cytoplasm of the cells, and in the cytoplasmic extensions, several types of microvesicles were also found to be empty or filled with electrodense material. At higher magnification, the tips of the cilia are enclosed by a striated membrane (Figure 6B) where a network of tiny microfibrils can be seen. The mesh was found to be inserted in the ciliary rootlets; in these structures there are basal bodies of cilia as suggested by observations of longitudinal and cross sections of TEM images (Figure 5D, 6B and Figure S1B). In Figure S1, non-canonical and canonical ciliary composition of two different levels of a cross sectioned flame are shown; at the level of the ciliary rootlets, axonemes with a MT distribution of 9+0, apparently corresponding to those well known for ciliary basal bodies (Figure S1B) while axonemes of other parts of the flame have a canonical 9+2 disposition (Figure S1C). In Figure S1C, axonemes appeared to be joined by tiny extensions while other similar connections can be seen between each cilium. Those connections were not seen in the cross section of basal bodies seen in Figure S1B and in the Figure S1A that corresponds to the FC from an adult parasite.

**Figure 7:**
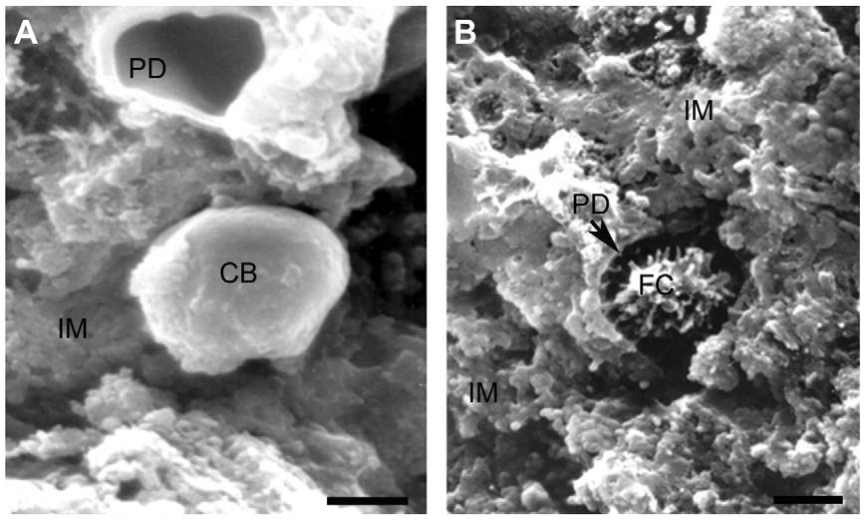
Scanning electron microscopy of a flame cell. The micrograph was taken from a cross-sectioned body of the proglottid of an adult tapeworm. In A, a cell body (CB) is proximal to the empty lumen of a protonephridial duct (PD). In B, the cilia of a flame cell (C) are close to extensions from the wall of the PD (arrow). In both images, flame cells (FC) are surrounded by a spongy interstitial matrix (IM). Scale bars A = 1 µm and B = 2 µm.

In observations by SEM of parenchymal tissues of one adult tapeworm, a FC was found (Figure 7): The parenchyma appears spongy and, inside of a parenchymal crater, a cell body is protruding (Figure 7A) while the ciliary tuft is inside of the excretory duct (Figure 7B). These images could correspond to the flickering cell beating seen at the movie S1. Near to the crater, there is an empty collecting duct and virtual immersions in these craters produced the impression they were connected with other big collecting ducts as it can be seen in the movie S6.

Micrographs of cilia cross-sections, taken at different levels of the flame and the cell body, depict similarities of ciliary patterns to those described in textbooks [20], [21], [22], [23]. Association of these images with those recovered by LSCM and TEM observations (Figure 2–5 and Figure S1) were used for designing an animation of a single *T. solium* FC as shown in movie S6. Similar drawings were published for other Platyhelminthes [1], [4], however in the present movie were added colors to indicate fluorescence markers observed in Figures 2 and 4. Using the animation, in order to illustrate the motions of the cytoskeletal proteins of a *T. solium* FC seen by TEM (Figure 5C) we are showing two possible ways for delivering substances to the cell body; one, inside of vesicles produced by invagination of the plasmatic membrane and two, for vesicular trafficking from extended processes of cells in communication. In the same animation, after the substances were delivered to the striated rootlet region (as suggested by TEM images in Figure 5A–C), their contents were discharged to the flame. After that, due the compression of the F-actin belt-clasp and the up and down vertical movements of the cilia, the substances can be throwing delivered to the collecting tubules and finally, they can be expelled trough the excretory system of the parasites. In the animated cartoon, the internalization of macromolecules could be related to the images of the Figure 5A and B as described earlier about the numerous microvesicles that are in apparent process of invagination or evagination; in the movie, a macromolecule (proposed as a globular protein) is taken by a membrane invagination, introduced in a microvesicle and transported through the cytoplasm until the fusion of the microvesicle with the striated rootlet region. In this region, the liberated macromolecule is diffusing trough the striated rootlet region to the lumen of the FC where the squeezing and the movement of the cilia redirect it to the protonephridial duct. Other solutes, considered of low molecular weight like electrolytes or organic compounds, could be taken by from the extended processes coming from other cells as suggested from Figures 5A, B and C and, because of the squeezing and the ciliar movements, these solutes can be redirected to the PS ducts for their elimination/reabsortion. Reabsortion could be executed by ducts trough the extensions seen at TEM in Figures 6A and by SEM in Figure 7B.

In order to combine all LCSM and TEM images and computational animation in order to present the animation in a 3D format, we decided to apply the 3D Max software on a *T. solium* FC image obtained by TEM (Movie S7). For this purpose, we took into account the observations of nuclei stained with DAPI (Figure 2), the co-expression of F-actin and myosin II (Figure 3), the 3D reconstruction of the F-actin clasp (Figure 4), the α-tubulin fluorescence staining (Figure 2) and images obtained by TEM and SEM (Figure 5, 6 and 7).

## Discussion

### Results of morphology, dynamics and computational visualization of flame cells of tapeworms of medical importance

As the present studies illustrate, a complete microscopic analysis and morphological characterization of *T. solium* cysticerci flame cells are described for the first time. The importance and implications of these studies can be extended to the cestoidea class and also, they can be applied to the Platyhelminth phylum. Better knowledge of the composition and the dynamics of FC could give a better understanding about the terminal cells of the complex excretory systems of these organisms [1]. In addition, for medically important helminths, this knowledge could contribute to better design of antihelminthic drugs since MT are the main cytoskeletal proteins for the functioning of the FC cilia and any alteration of them will produce malfunctioning of the excretory systems as was shown after *in vitro* treatment of *T. crassiceps* [12]. In order to survive inside the host, cysticerci need to carry out a continuous turnover of substances, for which the maintenance of an intact excretory system appears to be crucial [3].

Since the flame cells cannot be seen by direct observation of live intact cysticerci because the parasite tissue layers (brush border, syncytial tegument and myocyte fibers) impede their visualization. Punctured parasites were used to observe the flickering FC from the inner bladder wall where they were easily identified by the typical movements of beating cilia (Movie S1). The behavior of the ciliary tufts in FC was found to be similar to that described previously for *T. solium* [11] and other cestodes [4] and further indicates that these ciliary cells are highly dynamic. It is possible that the dynamics of the ciliary beating could be associated with the influence of the high content of calcium [22] that is stored in the calcareous corpuscles, mineral concretions found in the lumen of the channels of the cysticerci PS [19]. As suggested, by the presence of the flames in the collecting ducts (Figures 2C, 6A, Movie S1), the flickering movements could determine the flow of fluids inside of PS. Since FC are terminal cells of PS in invertebrates [1] their function is probably important for delivering substances either for elimination or reabsorption and, in tapeworms, they are thought to maintain the osmoregulation, helping to preserve the equilibrium between the hosts and parasités microenvironments [2]. The dynamics and the biological roles of FC require that they be interconnected with other types of cells that belong to the PS as found in the present TEM observations (Figure 6A) and the computational reconstruction (Movie S5). According with this, the FC need a specific size and morphology supported by their cytoskeleton [20], [21], [22], [23] that can be evaluated with the effects of specific substances that act on cytoskeletal proteins.

Previous descriptions of the cysticerci FC by light microscopy offered a static description of these cells [10], [11]. In the present studies, the use of videomicroscopy offers the possibility of linking the morphology with their dynamics in live parasites and their interactions with the excreting ducts (Movie S1). Integration of the real time visualization with fluorescence observations of the distribution of their cytoskeletal proteins (Figure 2) permitted reconstruction (Movies S3 and S4) of the internal morphology of these cells as seen in Figure 4. With the resolution using the Nomarsky optics it was possible to see the cilia of the flames (Figure 2I, Movie S1). By the use of electron microscopy combined with computational reconstructions new details of the previously described morphological analysis and, as seen in movie S6 and S7, produced animations of the structure and the cellular mechanics of a FC at the ultrastructural level. The combination of several microscopy methods is considered an excellent tool for presenting visual data [24], [25] in addition to the performance of the computer-based 3D reconstructions using AMIRA software [26]. More ultrastructural visualizations, like those produced by electron tomography [27], [28], could reveal more details of FC of these parasites.

### Cytoarchitectural distribution of cytoskeletal proteins in cilium-based cells

Detection of α-tubulin in cysticerci tissues indicates that this cytoskeletal protein is highly expressed in the syncytial tegument (Figure 1) and in many FC; the majority of these ciliated cells (approximately 80–90%) were found in tissues of the spiral canal of the invaginated scolex. Cestodes do not have a digestive system, so that the presence of tubulin is important for their survival and adaptation inside hosts, because the absorption-excretion-secretion system permits the continuous absorption of nutrients as well as an effective waste elimination [2]. The presence of α-tubulin in the syncytial tegument can be associated with requirements of MT that are necessary for maintaining the highly dynamic vesicular traffic required for uptake and secretion of macromolecules as demonstrated for *T. crassiceps* cysticerci [29] and are well known in eukaryotic cells [20]. However, for FC, the MT is necessary for the function of these ciliated cells [22] as described previously. However, since these cells are not easy to find in live parasites due their deeper tisular localization and the constant movements of the tissues; it could be useful to transfect the parasites with a GFP-tag for visualization of tubulin and to observe directly the dynamics of the FC as established for the study of the dynamic processes in ciliary movements [30].

As in the case of α-tubulin, F-actin is another cytoskeletal protein highly expressed in FC of *T. solium* cysticerci. The expression of the protein in similar cells was observed in other cestodes such as *D. dendriticum* [6], in the monogenean *G. rysavyi* [7] and the trematode *S. mansoni* [9]. In the former, it was found as part of a ring situated in the middle portion of their ciliated cells while in the later, with 3D reconstructions, it was found to be concentrated in a hollow barrel. It was interesting that phalloidin was found in in the belt clasp of *T. solium* FC (Figure 2E) and similar fluorescent patterns were observed for *D. dendriticum* [6] and *S. mansoni* [9]; as proposed for *S. mansoni* [9], the higher concentration of F-actin in this region could be related with the contractile motions of these cells. However, there may be an association with α-tubulin (see later) in order to maintain the cellular structure and the dynamics of the ciliary tuft.

Only co-localization of F-actin and myosin II were detected with high yellow fluorescent intensity in dots scattered in the IM as seen in Figure 3. Due the cluster of fluorescent dots contiguous to circular structures, presumably corresponding to cross-sectioned PD as described for flame cells after using the Bodiańs protargol method [10], the observation could suggest the presence of an actomyosin contractile system as found in contractile rings in cell divisions [31]. If the contractile system is located at the the actin clasp of *T. solium* FC (Figure 2), it suggests that myosin II is a linear molecular motor required for any constriction of the clasp as the proposed driving force in contractile rings [32]. Localization of F-actin with α-tubulin to find that the actin clasp is apparently embracing the ciliary tufts of the FC (Figure 2F–2H) and the distribution of these cytoskeletal proteins indicate that the actin clasp could be also associated with the MT of the flames. If this is a real situation, the role of MT at this site is for inducing the necessary movements of the suggested contractile system in order to favor propulsion of substances to the PD as inferred from the animated movie S7 and movie S1 where a FC is in direct contact with a PD. There is a microtubule-dependent regulation of the actomyosin system during the contractile ring assembly [31], however, this proposal needs to be characterized for the contractile machinery in *T. solium* FC.

In ultrastructural observations, at the level of the ciliary tuft and the cell body (Figures 5 and 6B), it was observed that these cellular structures were apparently fastened by a striated rootlet region in a similar fashion as described for ciliated cells [33]; the presence of a microfibrillar mesh distributed in an horizontal disposition appears to firmly surround the cilia tips (Figure 6B). One support for the biological role of this mesh could be the detection of γ-tubulin in this region because this is one of the most important proteins found inside of basal bodies and in the site of nucleation of ciliary MT [20], [21], [34]. Additionally, the structuring of the rootlet region that involves tiny microfibrillar strands and the ciliary rootlets (Figures 5D and 6B) could be important for anchoring the flame during the flickering motion as seen in the movies S1 and S2. It will be of interest to define the nature of these tiny microfibrils that could be coordinated with other cytoskeletal proteins that have a supportive function.

At the level of the longitudinal and cross sections of cilia of the Figures 5D and 6B, it was found, that 80 to 90 cross-sectioned axonemes were seen within cysticerci FC (Figure S1B and S1C) and this number could be useful for phylogenetic considerations as proposed for the phylum of Platyhelminthes [1]; in the cestode *Trilocularia acanthiaevulgaris* there are 50–70 axonemes [35] while 40–50 axonemes were found in *A cirrusspiralis* [1]. Apparently, as seen in more detail in the Figure S1B, there are tiny extensions that are required for joining each cilium of the *T. solium* FC cilia. Possibly, any disruption of these tiny structures could produce a failure of the FC functions and they need to be characterized.

The ultrastructure of *T. solium* flame cells appears similar to that of *A. cirrusspiralis* [1] (Figure 5 and 6). TEM images present the cells interacting with PS duct cells trough leptotrichs and ribs and, in SEM images, *T. solium* FC (Figure 7B) appeared to interact through their leptotrich structures with extensions of PS duct cells. In the SEM images of Figure 7, FC are embedded in the IM, near to excretory ducts or inside of them and apparently the tuft of cilia of the image on Figure 7B corresponds to that seen on Figure 6A by TEM. Both images could suggest how the fluids and their materials are flowing through the ducts as a result of the ciliary movements. Ultrastructural observations, suggest also that FC have the capacity to interact with other cells trough cytoplasmic extensions that are seen (Figure 5A–5D); apparently there are intercellular pseudopods that hold FC and PS duct cells together. Intercommunications between FC and other cells could be part of the tubular network system and the synctitial structure of the collecting channels and tubules described for cestodes [36] and it is a visual example of how they can be present at deeper parenchymal tissues as seen in the final part of the movie S6. Continuous synctitial layers in cestodes facilitate their exchange of nutrients or their capacity to respond immediately to changes in their microenviroment [2].

### 3D Reconstruction of the cytoskeletal protein distribution in flame cells

In order to understand the localization of the cytoskeletal proteins in the flame cells we used the integration of light, fluorescence and electron microscopy observations, together with a previous report that these cells have intense flickering movements of their ciliary tufts [5], the recording of them (Movie S1) and their computational visualization could be used as a approach for studying the functionality and the dynamics of these cells. Due the computational capability of reconstruction and resolution produced by the AMIRA software, we were able to produce a reconstruction of *T. solium* FC that allowed observation with an approximation to the ultrastructural level using a LSCM resolution. The 3D reconstructions of *T. solium* FC were achieved and visualizations were obtained that illustrate morphological details difficult to obtain by conventional TEM or SEM. For example, the F-actin in *T. solium* FC was found to be a cellular structure that looks like an belt clasp that is apparently embracing the ciliary tufts. The distribution of this actin clasp surrounds and encloses the ciliary tufts and apparently fastens the soma body. When the actin clasp is sectioned by computer, as shown in Figures 4C–E, and the 3D images are rotated in order to observe the center of the clasp, it was possible to see that there is a hole (See the animation presented in the movie S5). Co-localization of DAPI stained nuclei shows they appear to be positioned according to the plane of orientation of the actin clasp and the ciliary tuft. The meaning of these observations could be that the actin structure is necessary for holding the cell body to cilia. Also, it is possible that the morphological protrusion of the actin clasp, as seen in the reconstruction of the movie S5, could support the septate junction found between the extensions of the tubular cell interconnected cytoplasmic network as described for *A. cirrusspiralis* [1]. The AMIRA 3D volume reconstruction produced a solid actin clasp structure at the resolution of the LSCM; it will be necessary to know how the disposition of actin inside of the clasp is by evaluating its expression by immunogold assays for TEM or by the use of ET.

### Hypothetical visualization of the dynamics of cilium-based cells of the excretory system of tapeworms

It is clear that the results of this analysis show that FC contain at least three cytoskeletal proteins: F-actin, α-tubulin and myosin II, all proteins associated with contractile movements in cells. These cytoskeletal proteins are clearly located in the different structures of the FC. Flame cells are found in close contact or within the excretory system, suggesting that they are an integral part of the protonephridial system. However, whether these associations are involved in the flow of fluids in the protonephridial system, and their importance for survival in the host, remains to be established.

However, due in the present studies we were able to combine experimentally produced images from microscopy with computational imaging strategies; we consider that they can be integrated for producing visual dynamic images as presented in the movie S7 according with theorical knowledge of the studied cytoskeletal proteins. The animated model is an attempt for illustrating the functional activity of *T. solium* FC were the ciliated cells have intense movements of the ciliary tuft as seen in videomicroscopy (Movie S1). Entrance of the fluids in cells could be done trough the empty space in the clasp, where there is a connecting tubule of the tubular network, supported by the digital reconstructions of cross-sections images presented at Figures 4C–D. Inside of the cells, the fluids are pushed to the ciliary tuft due the contractibility/expansion of the actin clasp due to an acto-myosin system that produce a continuous squeezing of the ciliated cells (Movie S7) as produced during constriction of cells during the cellular division [20] and as the distribution of the cytoskeletal proteins was seen according with the 3D-volume reconstruction seen at Figures 2G, 2H and 4A–E. For macromolecules, their entrance to the cells could be performed by means of vesicular traffic; as suggested by electrodense vesicles seen at the soma bodies at TEM images (Figures 5A, 5B and 5C) and as presented in the animation of the movie S7; the macromolecules are uptaked into microvesicles seen in an apparent formation from plasmatic membrane invaginations, these microvesicles apparently travel inside the cytoplasm of the cell body and finally they are discharged trough the cross-striated rootlets region to the cilia. Later, because of the movements of the ciliary tuft, these movements drive out the contents into the lumen of the PS ducts. In this animated cartoon, we speculate on how solutes enter the cells, they pass through the FC and they are discarded to PS ducts. Similar experiments to those carried out with *T. crassiceps* cysticerci and schistosomula of *S. mansoni* for the uptake of macromolecules [29], [37] and the blocking of the internalization could offer support to the presented proposal. Resultant of combination of experimental and computational strategies, as performed in present studies, could be a valuable approach for trying to explain dynamic aspects and to think about the mechanical properties of ciliated cells as flame cells of the cestode *T. solium*.

## Supporting Information

Figure S1 Transmission electron microscopy of flame cell axonemes. Cross sections of the cilia of three flames are shown (A, B and C). Axonemes of FC from the invaginated scolex of a cysticercus that were cross-sectioned in two different regions are in A and B, while in C, there is an axoneme of a FC from an adult parasite. In A, axonemes in a distribution of 9+0 are presented and they were obtained near to the cross-striated rootlets region shown in figure 6B. In B and C, axonemes (white arrow) show a canonical distribution of 9+2 and they were obtained from regions localized at the level of the flames. Axonemes of cilia of cysticerci FC (B) appear to be more separated and connected by tiny prolongations (black arrowheads) that emerge from each cilium. In comparison, axonemes of adult parasite FC (C) appear to be with a closer interaction and delimited by hexagonal junctions. Observations were performed using a JEOL TEM at 60.0 KeV. Scale bars = 500 nm.
(0.91 MB TIF)

Movie S1 Flickering of a flame cell in a live cysticercus. Three presentations of flame cells are presented from time-elapsed sequences, under low magnification (40×) of live parasites seen under Nomarsky illumination conditions. First, inside of the parasite tissue, embbebed in the interstitial matrix, several FC could be seen moving. One flickering cell, surrounded by a red circle, shows their soma body oriented to the protonephridial duct. Later, in the optical zooming (14.8×) of the flickering cell, the flame shows some cilia signaled by white arrows. Finally, in the red circle, it is showing the beating of cilia of a FC inside of a protonephridial duct. The plane focus of the tissue is continuously moving due its contractibility (MPEG 4.14 MB).
(4.34 MB MP4)

Movie S2 Computational reconstruction of the F-actin in a flame cell. Z sequences of LCSM images were processed for their direct volume renderization with AMIRA software. In the movie, only F-actin (red) was reconstructed and its digital rotation show it looks like a clasp of a belt that fasten the ciliary tuft (green) (MPEG 8.48 MB).
(8.90 MB MPG)

Movie S3 Spatial projection of computational reconstructed F-actin clasps of several flame cells. After processing the images as indicated for the movie S2, the F-actin clasps of several flame cells are manipulated in order to observe how is their spatial volume and how this is associated with the embracement of the ciliary tufts (green) (MPEG 8.95 MB).
(9.39 MB MPG)

Movie S4 3D visualization and virtual immersion inside of the invaginated scolex. Z sequences of LCSM images as presented in figures 2G and 2H were computationally processed as indicated for figure 4 and movies S2 and S3. During the trip of the virtual immersion, from low to high magnifications and after performing approximations to FC, it is possible to distinguish how the ciliated cells are immersed in the interstitial matrix and how is visualized the topography of the reconstructed cytoskeletal components of the FC and the matrix (MPEG 6.67 MB).
(7.00 MB MPG)

Movie S5 Visualization of the co-localization of myosin II and α−tubulin in the invaginated scolex. After computational reconstructions, Z digital sections of the images permitted to observe the tisular distribution of muscle fibers (orange), the interstitial matrix (green), ciliary tufts emerging from the matrix, clasps of cells and several cytoplasmic extensions that interconnect cells. In the middle of the clasps, it was found that they have central empty holes (MPEG 1.21 MB).
(1.28 MB MPG)

Movie S6 Reconstruction and animation of cytoskeletal proteins of a flame cell. Using the TEM image of the figure 5C, according with the flickering of the FC shown in the movie 1, it was obtained a 3D Max reconstruction of the cell. For this, a combination of the figure 4 and movie S3 was done and as a resultant 3D reconstruction was obtained. In the movie, the hypothetical animation of the dynamics between the F-actin clasp (red) and the tubulin of the ciliary tuft (green) is showing the function of these cytoskeletal proteins of the ciliated cell (MPEG 5.76 MB).
(6.04 MB MPG)

Movie S7 Animation of the hypothetical function of flame cells. Using as a template the images from the figures 2, 4–7, animations of movies S3 and S4 and videomicroscopy of the movie S1, they were combined for performing a 3D animation by 3D Max software. In the animation, the contraction and expansion of the F-actin clasp and the flickering of the ciliary tuft is responsible for routing the contents of the vesicles (an orange globular particle) to the lumen of the protonephridial ducts. These contents could be come from invaginations at the membranes of the soma bodies or from the interconnections of other cells, the vesicles go through the cytoplasm of the cell bodies and they deliver their contents to the level of the actin clasp (MPEG 3.87 MB).
(4.07 MB MPG)
