# Supplementary figures and images for: Visualization and 3D Reconstruction of Flame Cells of Taenia solium (Cestoda)
Source: PLoS One. 2011 Mar 11;6(3):e14754. doi: 10.1371/journal.pone.0014754 (PMC3055865; doi:10.1371/journal.pone.0014754)

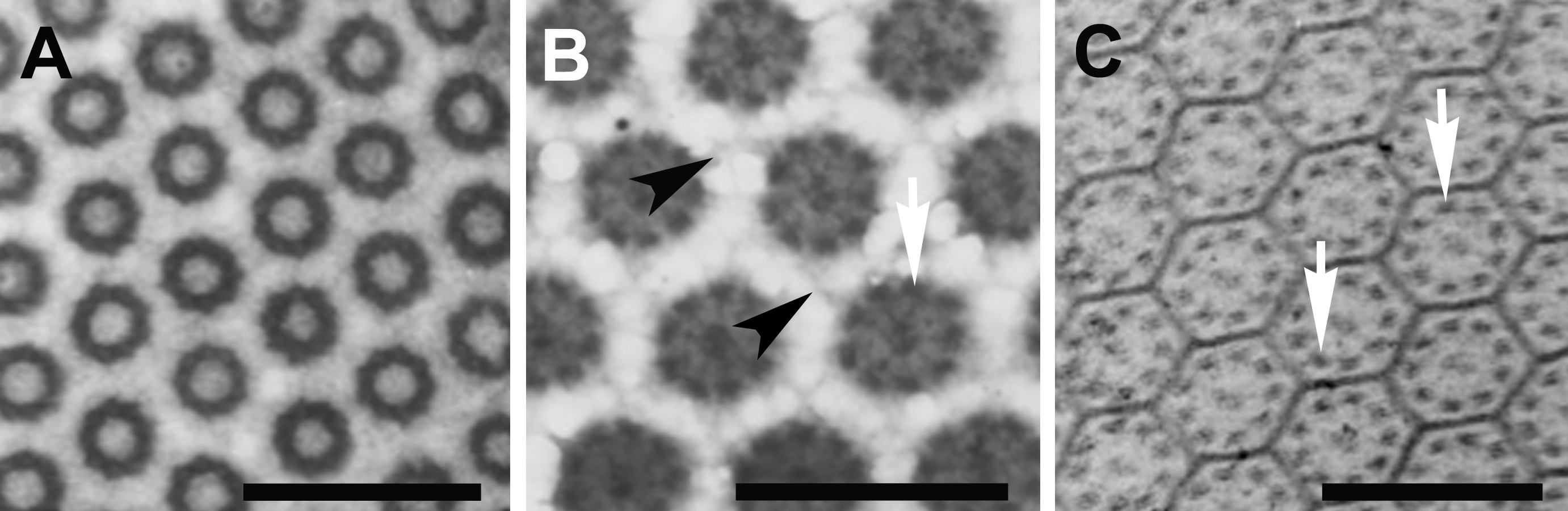

Supplement: Figure S1 — Transmission electron microscopy of flame cell axonemes. Cross sections of the cilia of three flames are shown (A, B and C). Axonemes of FC from the invaginated scolex of a cysticercus that were cross-sectioned in two different regions are in A and B, while in C, there is an axoneme of a FC from an adult parasite. In A, axonemes in a distribution of 9+0 are presented and they were obtained near to the cross-striated rootlets region shown in figure 6B. In B and C, axonemes (white arrow) show a canonical distribution of 9+2 and they were obtained from regions localized at the level of the flames. Axonemes of cilia of cysticerci FC (B) appear to be more separated and connected by tiny prolongations (black arrowheads) that emerge from each cilium. In comparison, axonemes of adult parasite FC (C) appear to be with a closer interaction and delimited by hexagonal junctions. Observations were performed using a JEOL TEM at 60.0 KeV. Scale bars = 500 nm. (0.91 MB TIF) [file pone.0014754.s001.tif]
